# Targeting and Crossing the Blood-Brain Barrier with Extracellular Vesicles

**DOI:** 10.3390/cells9040851

**Published:** 2020-04-01

**Authors:** Julien Saint-Pol, Fabien Gosselet, Sophie Duban-Deweer, Gwënaël Pottiez, Yannis Karamanos

**Affiliations:** 1 Laboratoire de la Barrière Hémato-Encéphalique (LBHE), UR 2465, University Artois, F-62300 Lens, France; fabien.gosselet@univ-artois.fr (F.G.); sophie.duban@univ-artois.fr (S.D.-D.); yannis.karamanos@univ-artois.fr (Y.K.); 2 Caprion Biosciences Inc., 141, Avenue du Président-Kennedy Suite 5650, Montréal, QC H2X3Y7, Canada; gpottiez@caprion.com

**Keywords:** blood–brain barrier, extracellular vesicles, exosomes, microvesicles, brain diseases

## Abstract

The blood–brain barrier (BBB) is one of the most complex and selective barriers in the human organism. Its role is to protect the brain and preserve the homeostasis of the central nervous system (CNS). The central elements of this physical and physiological barrier are the endothelial cells that form a monolayer of tightly joined cells covering the brain capillaries. However, as endothelial cells regulate nutrient delivery and waste product elimination, they are very sensitive to signals sent by surrounding cells and their environment. Indeed, the neuro-vascular unit (NVU) that corresponds to the assembly of extracellular matrix, pericytes, astrocytes, oligodendrocytes, microglia and neurons have the ability to influence BBB physiology. Extracellular vesicles (EVs) play a central role in terms of communication between cells. The NVU is no exception, as each cell can produce EVs that could help in the communication between cells in short or long distances. Studies have shown that EVs are able to cross the BBB from the brain to the bloodstream as well as from the blood to the CNS. Furthermore, peripheral EVs can interact with the BBB leading to changes in the barrier’s properties. This review focuses on current knowledge and potential applications regarding EVs associated with the BBB.

## 1. Introduction

The brain is protected from the periphery by different barriers including the blood-brain barrier (BBB) and the choroid plexus. Both of these barriers, having important protective roles, have been studied widely over the past few years. One of the current strategies for specifically targeting the BBB is to design vehicles able to cross the barrier using nanoparticles (NPs). The therapeutic compound can thus be enclosed inside a functionalized capsule made of lipids or polymers [1]. Such features allow the drug to be protected at many stages starting with its transport from the digestive tract to the blood stream. There are several parameters that affect the efficiency of nanoparticles systemic circulation, BBB passage and cellular delivery [2]. Among them are, notably: (i) a clear inverse correlation among NPs size and BBB penetration; (ii) the influence of NPs shape on body distribution and cellular uptake; (iii) the type of ligands; and (iv) the zeta potential. Moreover, with specific structures presented at the surface of the nanoparticles, it is possible to target and penetrate the brain, via transportation through the BBB. As we have recently noticed [3], the information found on the surface of extracellular vesicles (EVs) could be used to functionalize nanoparticles that could travel unharmed and reach the brain.

### 1.1. Brain Barriers

**Figure 1:**
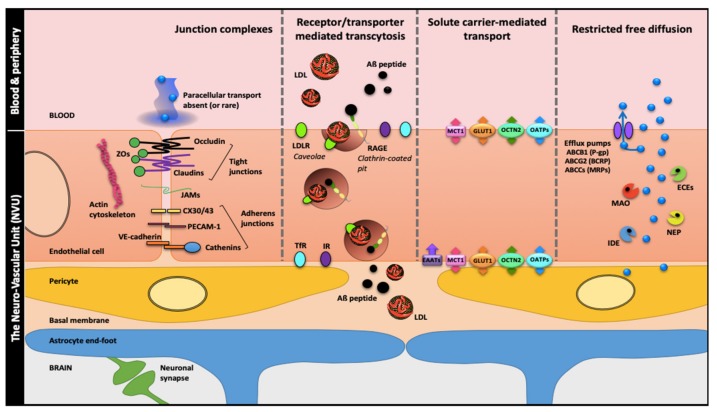
The blood-brain barrier (BBB), a solid wall within the brain microvasculature. Brain microvessels endothelial cells (ECs) are the bricks supporting the BBB phenotype with two main components: (i) a physical barrier which restricts transcytosis and seals the paracellular spaces between ECs through apical tight junctions (claudins, tricellulin and occludin linked to actin cytoskeleton by zonula occludens proteins), median junctional adhesion molecules (JAMs) and medio-basolateral Ca^2+^-dependent adherens junctions; (ii) a metabolic barrier supported by enzymes and efflux pumps restricting nonspecific transport and favor receptor or transporter-specific routes. These features which cement the BBB ‘wall’ are induced, organized and maintained by cell–cell communications between the ECs and the close neighboring cells: brain pericytes, astrocytes (through their end-feet surrounding the brain microvessels) and neurons. These four cell types together with the basal membrane form the neurovascular unit (NVU). Abbreviations: ABCB1: ATP-Binding Cassette sub-family B member 1; ABCCs: ATP-Binding Cassette sub-family C members; ABCG2: ATP-Binding Cassette sub-family G member 2; Aβ: amyloid-β; BCRP: Breast Cancer Resistance Protein; CX30/43: Connexin 30/43; ECEs: Endothelin-Converting Enzymes; EEATs: Excitatory Amino acid Transporter 2; GLUT1: Glucose Transporter 1; IDE: Insulin Degrading Enzyme; IR: Insulin Receptor; LDL: Low-Density Lipoproteins; LDLR: Low-Density Lipoproteins Receptor; MAO: MonoAmine Oxidase; MCT1: Monocarboxylate Transporter 1, MRPs: Multidrug-Resistance Proteins; NEP: Neprilysin; OATPs: Organic Anion Transporting Polypeptides; OCTN2: Organic Cation/Carnitine Transporter 2; PECAM-1 (also CD31): Platelet Endothelial Cell Adhesion Molecule-1; P-gp: P-glycoprotein; RAGE: Receptor for Advanced Glycation End-products; TfR: Transferrin Receptor; ZOs: Zonula Occludens.

The BBB is located in brain microvessels and is considered to be the major brain barrier in terms of length, close to 650 km, and surface, 10–20m^2^ [4]. This barrier maintains the brain homeostasis and protects the brain from exogenous and circulating threats. Despite decades of research and progress, the understanding of the BBB is not exhaustive and crossing it remains a major obstacle for new brain therapies. For a long time, especially when cell culture models were used, studies on BBB were focused on the contribution of endothelial cells alone. It is only recently that other cell types located in their vicinity in brain tissues, i.e., astrocytes and pericytes, have been added [5,6]. It is now admitted that the microvascular endothelium, glial cells, pericytes and neurons, and their intercommunication, constitute an assembly of cells (Figure 1). This cell assembly has more recently been named the “neurovascular unit” (NVU). Moreover, the extracellular matrix (ECM) or basal membrane is an integral part of the NVU. Considering the BBB in its environment, it is critical to understand the development of this barrier and its physiology [7,8]. The NVU and its dynamics are both keys to understanding how the different components act in concert to modify cerebrovascular function and permeability in health and in diseases [9]. Knowledge of the BBB, including its protein expression helps in the development of therapeutics targeting the NVU and thus the CNS.

The characteristics of the endothelial cells (EC) determine the BBB’s properties but are dependent on their communication with other NVU components [10]. The tight junctions (TJs) between the endothelial cells block the paracellular pathway. Thus, the decrease in the integrity of TJs result in inflammation, edema and neuropathologies [11]. The transcellular pathway could be mediated by channels, constituted by connexin or pannexin providing direct diffusion or indirectly by controlling the intracellular levels of Ca^2+^ [10]. This ion, involved in signaling, is associated with several steps in the vesicular pathway. In addition to TJs and Ca^2+^-dependent adherens junctions as summarized in Figure 1, the BBB phenotype is composed by efflux pumps such as P-glycoprotein and multidrug-resistance proteins (MRPs), some enzymes, and specific receptors and transporters for transcytosis routes we previously described for the low-density lipoprotein receptor (LDLR) and for the receptor for advanced glycation end-products (RAGE) for amyloid-β (Aβ) peptide entry from the bloodstream into brain [12,13,14,15]. Meanwhile, in therapeutic research, endothelial cells and astrocytes are considered to be two of the main actors of the BBB, the pericyte-endothelial crosstalk should be taken into account when designing cellular therapies [16,17], since brain pericytes are thought to be one of the main inductors of the BBB phenotype [18,19]

Other barriers such as the blood-cerebrospinal fluid (CSF) barrier comprising the choroid plexus (CP) of each brain ventricle and the arachnoid barrier (AB) cells in the meninges [20] protect the brain. However, considering that the focus of this review is the EVs and their relationship with brain barriers, BBB and CP are the only barriers reported in the literature in that regard. Studies of EVs contained in the CSF can reflect the physiology of the choroid plexus epithelium (CPE), as well as the intercommunication between barriers.

### 1.2. Extracellular Vesicles (EVs)

EVs are small membrane-bound particles that package and transport lipids, nucleic acids and proteins. Theoretically they are produced by all cell types. They are considered an additional mechanism for intercellular communication [21], allowing, for example, cells to exchange genetic material, lipids and/or proteins. Through such exchanges, cells communicate using EVs as "messengers". The term EV regroups a plethora of secreted, circulating vesicles that are in general described in a specific tissue and named in relation to their tissue origin or secretion pathway. Moreover, in the face of the rapid growth of studies on EV and exosomes (EXOs), the Minimal Information for Studies of Extracellular Vesicles (MISEV) provides recommendations for the nomenclature, collection and pre-processing, EV separation and concentration, EV characterization, functional studies and reporting. Indeed, such guidance is essential for characterizing the EVs’ roles better and avoiding attributing inappropriate functions to EVs subpopulations. The first MISEV was established in 2014 [22] and was updated in 2018 [23]. In addition to the MISEV, the EV-TRACK (Transparent Reporting And Centralizing Knowledge in extracellular vesicle research) consortium proposed a tool named EV-METRIC to improve experimental rigor following a quality chart in nine parameters for more transparence in the methods used for EV separation and characterization [24]. With these considerations, the present review gives some specifications about the EV classification as far as the materials and methods published in the quoted studies are precise and in agreement with the MISEV recommendations. Over the years, the list of functional generic markers characterizing EVs has been evolving as some teams have demonstrated their strength or irrelevance in EV studies. As an example, acetylcholine esterase function, was considered as a gold standard in EV characterization. It has recently been shown that this enzyme is not a generic marker of EVs as its activity seems to be related to a small subset of EVs [25].

EVs are gaining relevance due to their prominent function in intercellular communication among brain cells and involvement in a wide range of role with regard to physiology, pathology and therapeutics [26]. Indeed, EVs have been described as vectors in pathogenesis and in intercellular communication and could therefore be used for vaccine or drug delivery [27]. Biomarkers could be revealed among the content of EVs [28].The molecular data collected during studies on EVs were regrouped in three different databases: (i) Vesiclepedia (http://www.microvesicles.org/) ‘a manually curated compendium of molecular data (lipid, RNA, and protein) identified in different classes of EVs’ [27], from more than 1250 independent published and unpublished studies at the time this review is in writing; (ii) ExoCarta (http://www.exocarta.org/) [29], listing the identified contents of EXOs from multiple organisms; and (iii) EV-TRACK (http://evtrack.org/) using EV-METRIC not only designed as a tool to validate EV studies, but also to generate a wide public EV-TRACK knowledgebase from submitted and already published experiments [24].

**Figure 2:**
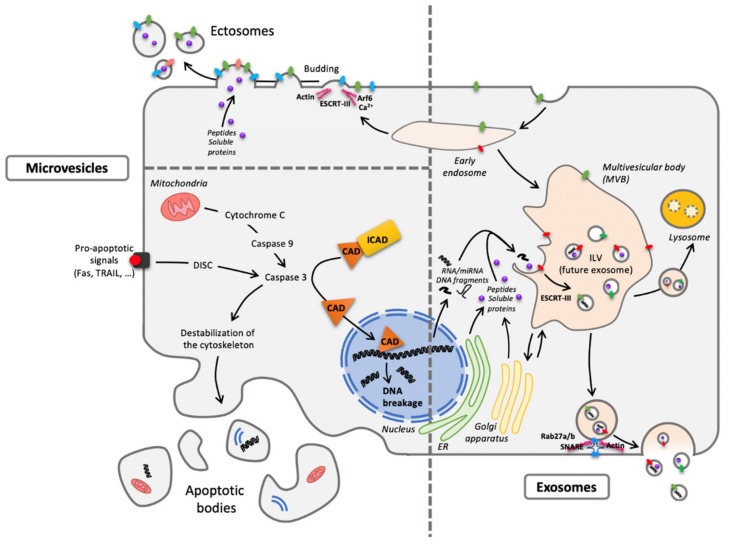
One cell, different extracellular vesicles (EVs). The two main forms are microvesicles (MVs) and EXOs differing from their origin. Concerning MVs, ectosomes result from membrane budding (left pane, top) and apoptotic bodies from cellular vesiculation following apoptosis (left panel, bottom). As to exosomes (EXOs), they have an endosomal origin, generated by the formation of intraluminal vesicles (ILV) within the multivesicular body (MVB) (right panel) and released through exocytosis routes. Despite a quite homogenous diameter from 30 to 150 nm, EXOs are difficult to distinguish (size exclusion chromatography or differential ultracentrifugation) from the smallest MVs. Abbreviations: CAD: Caspase-Activated DNAse; DISC: Death-Inducing Signaling Complex; ESCRT: Endosomal Sorting Complexes Required for Transport; ER: Endoplasmic Reticulum; ICAD: Inhibitor of Caspase-Activated DNAse; Rab: RAS-related protein; SNARE: Soluble N-ethylmaleimide-sensitive-factor Attachment protein REceptor; TRAIL: Tumor-necrosis-factor Related Apoptosis Inducing Ligand.

Little is known about the composition and functional differences between EVs subsets such as (i) EXOs, (ii) microvesicles/shedding particles released directly from the plasma membrane in living cells, and (iii) apoptotic bodies released from the plasma membrane in dying cells. With respect to EXOs, they represent vesicles that are released to the extracellular environment after fusion of late endosomes/multivesicular bodies (MVB) with the plasma membrane. The biogenesis of EVs was extensively reviewed [30] and summarized in Figure 2.

The relationship between the phenotypic state of the cell and the content of the produced EVs is unclear [31]. The ‘information’ enclosed in these vesicles takes the form of patterns of proteins, lipids and nucleotides that omics tools help decipher and comprehend [32]. However, the identification of specific markers remains quite complicated due to the heterogeneous population of EVs and particularly small EVs. According to Théry’s team, which carried out a wide study to isolate the different populations of small EVs, EXOs present tetraspanins (CD9, CD63, CD81), syntenin-1, TSG101, and ADAM10 [21,33]. Moreover, EXOs are highly enriched in cholesterol, sphingomyelin, and hexosylceramides at the expense of phosphatidylcholine and phosphatidylethanolamine [34]. The surface information of the vesicle referred to as the EV signature could be employed for targeting the brain with nanoparticles or free drugs. For more information on EVs and their properties, we recommend referring to reviews [21,35], which provide a comprehensive description of EVs, their cell biology and their physiological functions.

Proteomics have been used to characterize EVs’ proteome, but many challenges have yet to be addressed [36]. This is particularly true in the specific case of the BBB. EVs have interesting properties that have not yet been thoroughly explored. Undoubtedly, EVs have the ability to interact with the endothelial cells, the first line of defense of the brain. Such interactions, mediated by surface markers, allow, in some cases, EVs to cross the BBB or modify its properties. EVs are also the way for NVU cells to respond to various environmental stimuli. We present herein the recent discoveries made with respect to brain barrier-associated EVs.

## 2. Communication with the Brain Barriers Involving EVs

The molecular signatures for brain endothelial cell-specific EVs under various biological conditions were recently identified [37], offering a potential source of useful biomarkers and eventually new receptors, that should facilitate delivery of compounds. 

Under normal or healthy conditions, shotgun proteomics and parallel reaction monitoring (PRM)-based mass spectrometry analysis on endothelial cell-derived EVs has demonstrated that MVs contained enriched amounts of mitochondrial and cytoskeletal proteins, whereas EXOs enclose more adhesion, histone and ribosomal proteins [31]. Since neurodegenerative disorders, neuroinflammatory responses and pro-inflammatory cytokines such as tumor necrosis factor α (TNFα), have been ascribed to promoting BBB leakage [38], this is ascribed to or thought to be linked with an EV-mediated process. The same study showed that several proteins involved in TNFα and NF-κB signaling pathways, found to be differentially expressed in cells, were also differentially expressed in both MVs and EXOs after TNFα treatment [31]. Thus, some identified proteins in normal and pathological contexts could be used to discriminate MVs from EXOs, and cells tend to systematically “package” proteins in EVs in accordance with their phenotypic state.

Viral infections can also influence the release of EVs from BBB endothelial cells [39]. Using a human cerebral microvascular endothelial D3 cell line (hCMEC/D3, model described in [40]) it was shown that HIV-1 increases the release of EVs, as well as elevating their amyloid-β (Aβ) cargo, when compared to non-HIV controls. The accumulation and the deposition of Aβ peptides in the brain of patients with Alzheimer’s disease (AD) leads to the production of amyloid plaque which is one of the hallmarks of the disease and is recognized as playing a crucial role in the central and neurovascular pathophysiological events of the disease. Interestingly, brain endothelial cell-derived EVs transferred Aβ to astrocytes and pericytes and successfully transferred across the BBB into the brain [41]. Based on these observations, the authors concluded that HIV-1 facilitates the shedding of brain endothelial EVs carrying Aβ, a process that may increase Aβ exposure of NVU cells and contribute to central and perivascular amyloid deposition.

The proteomic analysis of EVs in human CSF [42] concluded that several exosomal markers were enriched including alix, syntenin-1, heat shock proteins and tetraspanins. In addition, the EVs contained the amyloid precursor protein, prion protein and DJ-1, biomarkers for neurodegenerative diseases. Hence, the authors postulated that α-synuclein (α-syn) and Aβ peptides could be involved in the dissemination of the disease to other parts of the brain [42,43,44]. In AD, the composition of the EVs from astrocytes seems to be altered by both Aβ peptides/oligomers and tau proteins [43,45], highlighting the possible role of EVs as biomarkers since their composition reflects the pathological features of the disease.

The EV-mediated cell-to-cell communication is also related, to some extent, to control of or impact on the BBB phenotype. In a zebra fish model, it was established that brain-derived EVs and particularly EXOs holding the miRNA miR-132 can control the expression of an adherens junctions (AJ)-related protein (i.e., Cadherin 5 or VE-Cadherin), and the inhibition of miR-132 containing EXOs led to an increase in BBB permeability and microhemorrhage events along the brain microvasculature [46]. In another study, EXOs of pericytes were transplanted into mice with spinal cord injuries (SCI) to study the restoration of motor function and explore the underlying mechanism. Among others it was found that treatment with EXOs could improve endothelial function and it was suggested that one of the underlying mechanisms may be the protection of endothelial cells in hypoxia conditions [47]. The findings suggested that pericytes-derived EXOs was a potential new therapeutic intervention for SCI.

The release of EVs by the CPE was described as another mechanism of blood–brain communication. An interesting study [48] showed that peripheral lipopolysaccharides (LPS)-stimulated inflammation affects the choroid plexus and subsequently modifies the properties of the BBB. The CPE cells sense and transmit information from the peripheral inflammatory status to the CNS via the release of EVs into the CSF. The pro-inflammatory message is subsequently transmitted to recipient brain cells. Peripheral administration of LPS provokes inflammatory conditions that enhance the production of α-syn rich EVs by erythrocytes that can cross the BBB. Furthermore, these peripheral EVs might contribute to or even initiate CNS α -syn related pathophysiological events since they demonstrated an enhanced CNS response in comparison with the erythrocyte-derived EVs from healthy patients [49].

## 3. Transport of EVs

**Figure 3:**
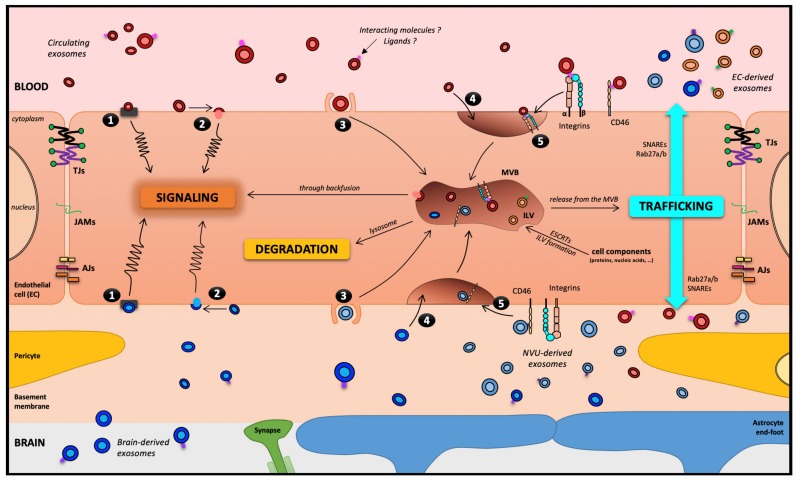
The blood–brain barrier, theoretically a motorway for a melting-pot of EXOs. Five routes have been described for EXOs interacting with a receiving cell: (**1**) association with a protein G-coupled receptor on the cell surface, inducing a signaling cascade; (**2**) adhesion to the cell surface and fusion, releasing the EXOs content in the cytoplasm, which can lead to several types of events, including cell signaling; (**3**) macropinocytosis; (**4**) nonspecific/lipid raft; or (**5)** receptor-mediated transcytosis, leading to its entry into the cell through the endocytic pathway and its storage in the MVB. Then, three outcomes remain possible for EXOs: (i) degradation by lysosomes; (ii) signaling induction through a backfusion event in the MVB releasing its content in the cytoplasm; or (iii) trafficking from the MVB to the plasma membrane as neoformed ILVs in the receiving cell. It is worth noting that except in pathological models, under TNF-α treatment [50], EVs and EXOs have not been described to cross the BBB through the paracellular pathway. Abbreviations: AJs: Adherens Junctions; EC: Endothelial Cells; ESCRT: Endosomal Sorting Complexes Required for Transport; ILV: IntraLuminal Vesicles; JAMs: Junctional Adhesion Molecules; NVU: Neuro-Vascular Unit; SNARE: Soluble N-ethylmaleimide-sensitive-factor Attachment protein REceptor; TJs: Tight Junctions.

As previously mentioned, circulating EVs can cross the BBB in both directions, i.e., from the bloodstream toward the brain, and from the brain to the bloodstream where it is easier to detect them. However, the transport routes taken by these EVs remain obscure, and the impact of EVs on ECs is also unclear. To date, focusing more specifically on EXOs, five theoretical routes have been highlighted to describe the interaction between EXOs and the receiving cell (Figure 3). Using an in vitro BBB model, Zhao’s team described that HEK293-derived EXOs exposed to the apical side of ECs can enter the cell through three different endocytosis processes: receptor-mediated transcytosis, lipid raft-mediated and micropinocytosis. The work also evaluated the EXOs fate. However, following transcytosis, their fate remains unknown [50]. More recently, using a sulfo-SBED (sulfosuccinimidyl-2-[6-(biotinamido)2-(p-azidobenzamido) hexanoamido] Ethyl-1,3- dithiopropionate)-based receptor collection method, Terasaki’s team identified potential candidates for the reception of EXOs on an EC surface such as integrins (more precisely α5 and αV) and CD46, a cluster of differentiation described as an adenovirus receptor. Indeed, the reduction of CD46 expression by RNA interference decreased the EXOs transport across the ECs 2-fold [51]. The candidates on the EVs surface are as numerous as the variability of their composition is great and associated with the EV-producing cell type. However, EVs present integrins and tetraspanins and some ligands for specific receptors for the receiving cell (such as the Transferrin for Transferrin Receptor on ECs).

Based on the hypothesis that brain cells, and more specifically neurons, would shed EVs that could be detected and purified, Zhang and coworkers established a method for extracting EVs with the surface marker L1 cell adhesion molecule (L1CAM) from blood [52]. The antibody-based extraction of EVs allows the quantification of biomarkers such as α-syn, which is more abundant in EVs extracted from Parkinson’s disease patients than healthy controls. Since then, several studies have been performed, using similar strategies giving new insight into the mechanisms by which the brain and periphery communicate during pathogenesis mediated by α-syn [49]. This strategy has also been extensively and successfully applied for the discovery of early markers in the blood of AD patients [53,54,55,56,57,58,59,60,61]. As an example, levels of P-S396-tau, P-T181-tau and Aβ1-42 in extracts of neurally derived blood EXOs predict development of AD up to 10 years prior to clinical onset [53]. The mechanisms of multiple sclerosis (MS) pathophysiological processes have not been fully elucidated, particularly those underlying the initial steps of the corresponding immune defects. It has recently been proposed that a significant contribution to the immune-regulatory events may derive from a cell-to-cell communication system involving the production, secretion and transfer of EXOs [62] and that their contents may serve as biomarkers.

All types of tumor-cell-secreted EVs can cross the disrupted BBB into the bloodstream [63], and thus represent potential biomarkers to improve diagnostics and follow-up for both low- and high-grade gliomas. The study conducted on mice evaluating EVs secreted from brain tumors was really informative [64]. First, the results showed that certain brain tumors may breach the BBB leading to an inevitable leakage of tumor’s EVs in the blood. Second, for tumors that do not affect the BBB, it is possible to quantify specific markers proving the crossing of EVs from the brain to the blood. This provides clues for future research regarding the diagnosis of brain tumors. 

The communication through EVs was also illustrated in the study of transendothelial migration; however, the underlying mechanism is subject to debate. A study carried out in 2016 [65] analyzed this phenomenon and showed that during experimental autoimmune encephalitis or from TNF-α-stimulated BMEC cultures, EVs cargoing CLN-5 protein can bind to leukocytes in vitro, highlighting the possible transfer of a TJ protein from endothelial cells to leukocytes through EVs leading to their transendothelial migration (Paul et al., 2016). Based on this discovery, new light is shed on this mechanism but also shows that more needs to be known about EVs as a way of communication and cell–cell interaction

## 4. EVs as Therapeutic Means

Three main approaches have been described to facilitate the passage of a therapeutic compound into the brain by crossing the BBB. These are referred to as invasive, pharmacological and physiological approaches (for review, see [66]). Invasive approaches deliver the active compounds by breaching the BBB. The pharmacological approach is based on the modification of active compounds and/or their formulation in order to give them attributes allowing their entry into the brain passively. The physiological approach benefits from the occurrence of endogenous receptors that are highly expressed at the surface of the BBB, for example, transferrin and insulin receptors. The author of the review describing these approaches concluded that the physiological approach seemed to be the most efficient one for obtaining a regular distribution of compounds in the brain after the transport of molecules through the endothelial cells of the BBB [66].

The interest in using EXOs as therapeutic delivery agents is growing, since they have a critical advantage in that they can cross the BBB, but surprisingly little is known about the mechanistic details of this process [67]. 

A method for non-invasive in vivo neuroimaging and tracking of EXOs was recently established [68]. EXOs were labeled with glucose-coated gold nanoparticles (GNP) and their uptake is mediated by the glucose transporter GLUT-1. The in vivo neuroimaging was enhanced when intranasal administration was used, due to a better brain accumulation. According to the authors, this approach can promote the study and application of EXOs-based therapies for brain pathologies [68]. A method was described to transport anti-inflammatory drugs, e.g., curcumin, to the brain using EVs released from several different cell lines-3T3L1, 4T1, CT26, A20 and EL-4-as a carrier method. The intranasal administration proved to be fairly efficient and led to a successful delivery of the active compound into the brain. The microglial cells were identified as being preferentially targeted by EVs [69,70].

Targeted EXOs were used to deliver siRNA to the brain in mice [71]. The authors used self-derived dendritic cells for the production of EXOs. The targeting capabilities were obtained by fusion of the rabies viral glycoprotein (RVG) peptide to the extra-exosomal N-terminus of Lamp2b. Purified EXOs were loaded with exogenous siRNA. As a result, the therapeutic potential of EXOs-mediated siRNA delivery was confirmed by the strong mRNA and protein knockdown of BACE1- a therapeutic target in AD-in wild-type mice. EXOs from dendritic cells have been largely used (i) for immunotherapy of primary brain tumor, (ii) as delivery vehicles of anti-tumor nucleotides, and (iii) as drug delivery vehicles for primary brain tumors [72]. It was also proposed that EXOs should be customized to deliver either an encapsulated drug or a signal worn by a surface protein. This bioengineering approach will be all the more attractive considering that some specific way of cell–cell interaction would be highlighted. This perspective was investigated for cancer immunotherapy, but it could also be attractive for the delivery of drugs into the brain. As a result, using EXOs for cancer immunotherapy was highlighted as a potent biological therapeutic [73].

Moreover, the use of the L-domain pathway has been suggested for the loading of exogenous proteins into EXOs [74]. Late or L-domains are sequences found in the group-specific antigens of retroviruses and are involved in the later steps of the virus budding process. These L-domains are highly conserved motifs known to mediate protein-protein interactions between cells [75] or within the cell such as described for syntenin and ALIX through the syntenin LYPX(n)L motif to promote EXOs budding and, therefore, their biogenesis [76]. Sterzenbach and colleagues used a WW-tagged Cre recombinase to interact with the L-domain of Ndfip1, an L-domain-containing protein known to be involved in the loading of protein into EXOs. The customized EXOs were delivered through the non-invasive nasal route to Ai14 mice. These mice were used as a control for brain-targeted Cre recombinase activity, and activated the tdTomato reporter in brain. This study demonstrated a way to deliver customized EXOs that were able to bring a protein into the brain or to act on targeted cells. Of course the translocation pathway is still unknown but the results are consistent with trafficking along perineuronal and/or perivascular channels [74].

## 5. Conclusions

Advances in research aiming at understanding EVs with regard to the BBB are extremely promising on several levels. First, they will allow an even more in-depth understanding of the BBB, namely (i) cell-cell communication strategies to promote and maintain the BBB phenotype; and (ii) the EVs’ transport routes through the ECs for the delivery of customized circulating EVs, putative therapeutics and their exit from the brain, which could be referred to as biomarkers in pathological contexts. Second, the use of EVs as carriers of biomarkers is clearly an advantage, and they were even called “a potential window to the brain” [77] and “goodies for the brain” [78]. In addition, last but not least, the use of EVs as transporters for targeting the brain is an appealing method for carrying therapeutics to their target. Studies have successfully shown valuable strategies for targeting the brain, as well as loading the therapeutic compound into the EVs [79]. Nevertheless, using EVs as carriers for therapeutic compounds is challenging [80], and more light needs to be shed on the specific receptors involved in the transport as well as the interaction with the target cells or tissues. Indeed, a complete understanding of the surface marker required to cross the barriers protecting the brain and the ones needed to target the cells or tissues responsible for a pathology are unmet needs in this field. Finally, in comparison to “uncovered” therapeutics, compounds enclosed in EVs will reach the brain more easily, and thus will have a preferred interaction with the BBB.
